# Global research and scientific publications on PND between 1969 and 2022: A bibliometric analysis

**DOI:** 10.1002/agm2.12310

**Published:** 2024-06-14

**Authors:** Ruoxuan Liu, Duan Gao, Ning Yang, Yu Qiao, Zihang Zhang, Mingzhang Zuo

**Affiliations:** ^1^ Department of Anesthesiology, Beijing Hospital, National Center of Gerontology; Institute of Geriatric Medicine, Chinese Academy of Medical Sciences. P.R.China Graduate School of Peking Union Medical College Beijing China; ^2^ Peking University School of Basic Medical Sciences Beijing China; ^3^ Shanghai Jiao Tong University Minhang Campus Shanghai China; ^4^ Department of Anesthesiology, Beijing Hospital, National Center of Gerontology; Institute of Geriatric Medicine Chinese Academy of Medical Sciences Beijing China

**Keywords:** bibliometrics, CiteSpace, perioperative neurocognitive disorders, scientometrics, VOSviewer

## Abstract

**Objectives:**

We hope to offer a comprehensive understanding of the advancements and patterns in research on PND. Methods: We performed a thorough search on the Web of Science Core Collection to locate relevant studies published from 1969 to 2022 and utilized four distinct tools, namely VOSviewer (J Data Inf Sci, 2017, 2, 1; J Am Soc Inf Sci, 1973, 24, 265; Amer Doc, 1963, 14, 10 and Scientometrics, 2010, 82, 581), CiteSpace (Scientometrics, 2010, 84, 523), Scimago Graphica, and R‐bibliometrix which allowed us to examine various aspects. Results: We included a total of 6787 articles and reviews for analysis which described PND research, the sources, and the subfields; highlighted the significant developments in this field; identified three main directions in PND.Conclusion: This study highlights the rapid growth of research on PND in recent years and provided an overview of previous studies in the field of PND, thereby establishing the overall landscape of PND research and identifying potential avenues for future investigations.

**Methods:**

We performed a thorough search on the Web of Science Core Collection to locate relevant studies published from 1969 to 2022. To perform bibliometric analysis and network visualization, we utilized four distinct tools, namely VOSviewer (J Data Inf Sci, 2017, 2, 1; J Am Soc Inf Sci, 1973, 24, 265; Amer Doc, 1963, 14, 10 and Scientometrics, 2010, 82, 581), CiteSpace (Scientometrics, 2010, 84, 523), Scimago Graphica, and R‐bibliometrix. These tools allowed us to examine various aspects, including the yearly publication output, the contribution of different countries or regions, the involvement of active journals, co‐citation analysis, publication status, keywords, and terms, as well as scientific categories. We hope to offer a comprehensive understanding of the advancements and patterns in research on PND. The insights gained from this study can assist researchers and clinicians in enhancing the management and implementation of their work in this field.

**Results:**

In this study, we included a total of 6787 articles and reviews for analysis. First, publication trends and contribution by country analysis described PND research. Second, a historical analysis described PND research, the sources, and the subfields. Third, an analysis of keywords highlighted the significant developments in this field. Fourth, an analysis of research themes identified three main directions in PND.

**Conclusion:**

In summary, the research volume exhibits exponential growth over time. Furthermore, the majority of contributions originate from Western countries and China. The interdisciplinary nature of the field is evident, with its roots in biology and medicine and further branching into psychology and social sciences. POCD, delirium‐predominant associated clinical management were major research themes about PND.

## Graphical abstract

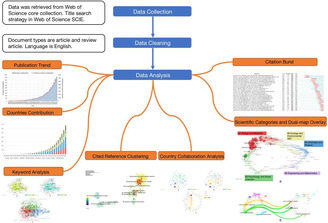

## 1 INTRODUCTION

Perioperative neurocognitive disorders (PND), also referred to as perioperative NCDs, are frequent neuropsychiatric complications observed in elderly patients following surgical procedures.^1^ These disorders are associated with increased morbidity, mortality, and high medical expenses. PND is an overarching term encompassing cognitive impairment identified either prior to or after surgery. This includes pre‐existing cognitive decline (termed neurocognitive disorder), any form of acute event (postoperative delirium (POD)), cognitive decline diagnosed within 30 days after the procedure (delayed neurocognitive recovery), and cognitive decline diagnosed within 12 months after surgery (postoperative neurocognitive disorder).^1^

The exact inception of scientific research on PND remains unclear; however, only a small number of studies were conducted before 1969. One noteworthy study by Morse, RR and Litin, EE et al. focused on POD, which served as the seminal investigation on PND.^2^ Subsequently, research on PND has gained momentum and flourished since approximately 2004. The annual publication output has consistently exceeded five articles since 2004, exhibiting an overall exponential growth trend. Consequently, a substantial body of literature on the topic has accumulated over time.

To gain insights into the structure of the research domain and identify significant trends and gaps, a mapping review methodology proves more suitable. Unlike traditional narrative literature review approaches, bibliometric analysis techniques employ quantitative and statistical methods to uncover high‐level patterns in the research domain. Utilizing bibliometric analysis and related visual analytics software offers appropriate methodologies for such investigations. Bibliometrics, an academic field pioneered by Pritchard^3^ and subsequently advanced by Cole and Price,^4^ employs quantitative analysis to investigate the characteristics of scientific endeavors. Through visual analytics approaches that incorporate processed bibliometric data, the research domain can be effectively examined, yielding insights into its structure.

**FIGURE 1:**
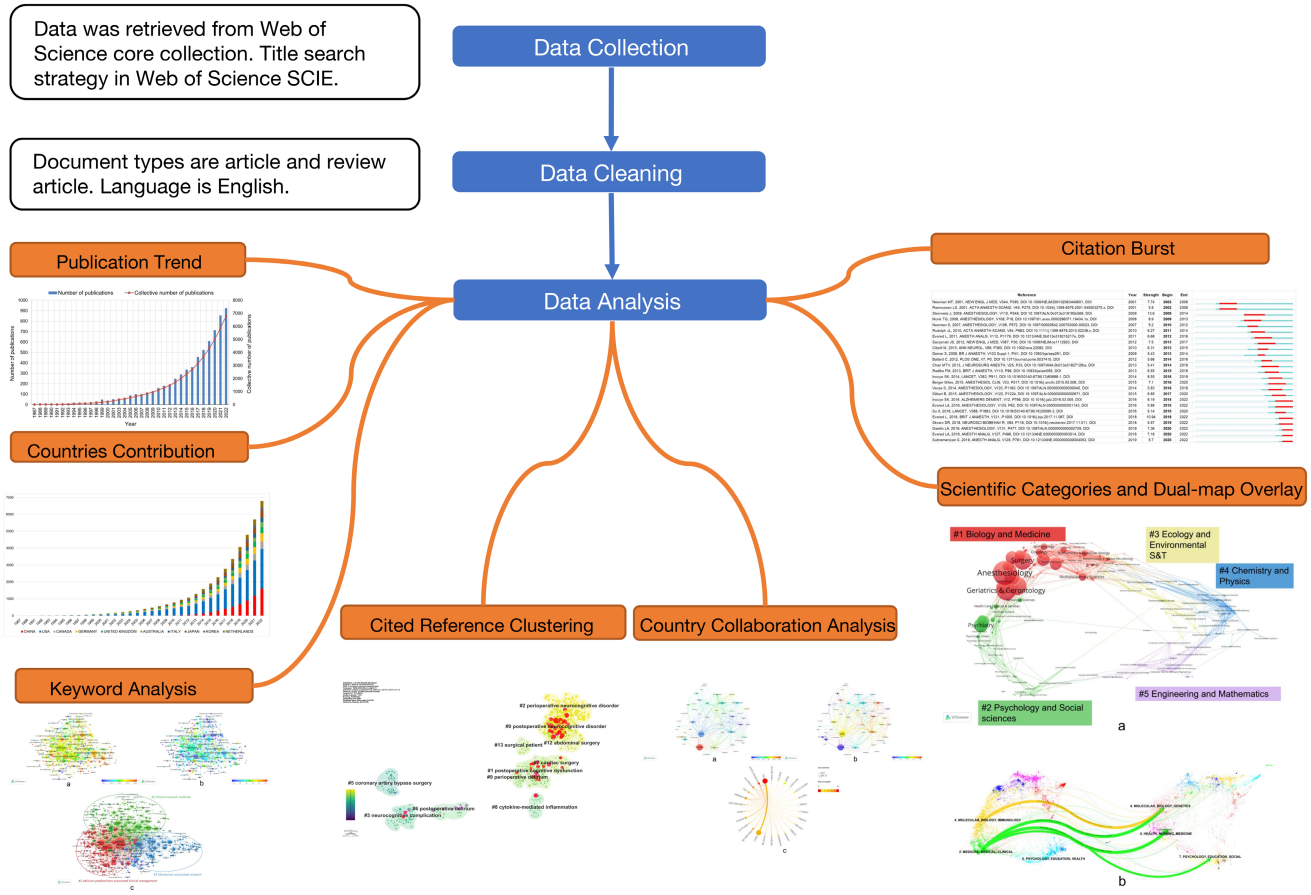
Flow chart.

Despite the extensive literature on postoperative cognitive dysfunction (POCD) since the 1970s, no previous bibliometric study specifically focused on PND has been reported. This paper aims to bridge this gap by providing a comprehensive overview of the developments and distribution of knowledge within the PND research domain. To achieve this, we conducted a search for articles and reviews related to PND in the WOS database. Descriptive and bibliometric analyses were performed to elucidate the knowledge base, hotspots, and prominent domains in the field of PND. Furthermore, we endeavored to uncover the evolution and future trends of the entire field by employing timeline analysis and burst detection of co‐cited documents. Through our study, we aspire to furnish researchers and clinicians with valuable insights and a better understanding of PND research, aiding them in planning and managing future work. We hope that our findings hold significant value, contributing to the advancement of PND research, while providing suggestions and inspiration for researchers and practitioners involved in the study and treatment of PND (Figure 1).

- Overall publication trends.
- Geographic trends at a country level.
- Major scientific categories.
- Major journals focusing on PND.
- Dominant narrative topics and their temporal evolution.
- Evolution in research clusters, associated research fronts, and key documents.

## 2 METHODS

### 2.1 Data sources and search strategies

The scientific literature on PND was retrieved from the online version of the Web of Science Core Collection, specifically the “Science Citation Index Expanded (SCIE)”. The Web of Science Core Collection is a prominent citation database that provides comprehensive bibliographic information for publications across various scientific disciplines. It has a rich history and offers high‐quality data and literature indexing, making it one of the most widely used databases for bibliometric research.

The search strategy we used was that TS = (“Cognitive Complications” OR “Cognitive Complication” OR “Cognitive Dysfunction” OR “Cognitive Dysfunctions” OR “delirium” OR “neurocognitive disorders”) AND TS = (“Postoperative”), document types were restricted to article or review article, and language was restricted to English articles. The search encompassed the entire available timeframe covered by the database. Initially, over 8000 publications were retrieved based on the defined search criteria. Subsequently, the results were refined by considering the Web of Science Categories associated with the publications. This refinement aimed to exclude articles from irrelevant categories, such as meeting summaries or letters, resulting in a more focused list of publications. Ultimately, a total of 6787 relevant publications were downloaded and analyzed for the present study. It is important to note that a single paper may be assigned to multiple categories within the Web of Science Categories. As a result, the sum of the papers within each category may exceed 6787, the total number of publications included in the analysis.

### 2.2 Bibliometric analysis

In this study, bibliometric mapping methods and tools were employed to visualize scientific data through various processes. Bibliometric mapping is a research approach that utilizes quantitative methods to visually represent scientific literature by leveraging bibliographic data.

### 2.3 Bibliographic analysis using VOSviewer (1.6.18)

Step 1: Download and install the VOSviewer software from the official website (https://www.vosviewer.com/).

Step 2: Prepare the data in the form of bibliographic records. Export the data in TXT format, including “full records and references.” The exported document records should include the title, author, research institution name (such as Research Institute, school, or hospital), abstract, journal, publication date, and other relevant information.

Step 3: Import the prepared data into VOSviewer.

Step 4: Configure the analysis parameters to customize the analysis. Adjust the number of documents associated with the nodes based on the data visualization requirements, while leaving the remaining parameters at their default values.

Step 5. Run the analysis:

1. Collaboration among countries or regions: We conducted a co‐authorship analysis of countries and regions to identify the key contributors in the field of sarcopenia.
2. Analysis of keywords and terms: The size of the nodes representing keywords is proportional to their frequency of occurrence. The thickness of the links indicates the strength of co‐occurrence between two keywords.
3. Scientific categories: Each journal was classified into specific scientific categories, providing insights into the disciplinary and domain focus of the journals.

### 2.4 Bibliographic analysis using CiteSpace (6.1.2)

Step 1: Download and install the CiteSpace software from the official website (https://citespace.podia.com).

Step 2: Prepare the data by exporting it in TXT format, including “full records and references.” The exported document records should include the title, author, research institution name (such as Research Institute, school, hospital), abstract, journal, publication date, and other relevant information.

Step 3: Import the prepared data into CiteSpace, selecting the appropriate file type and encoding. Check for any duplicate data before proceeding.

Step 4: Configure the parameters in CiteSpace as follows: (1) Set the time slicing from January 1969 to December 2022, with a time cutoff point of 1 year for analysis; (2) Select country, institution, author, and keyword as node types for visual analysis and generation of co‐occurrence maps; (3) For pruning, utilize the Pathfinder and pruning sliced networks in the pruning function area to emphasize the connection diagram of significant nodes. Leave the other parameters at their default values.

Step 5: Run the analysis, allowing CiteSpace to process the data and generate a network visualization based on the selected parameters.

1. Document co‐citation analysis: In this analysis, different cited references were depicted as distinct nodes. The size of the tree rings represented the citation history of the references, while the purple ring indicated high betweenness centrality, signifying strong connections with other nodes or central positioning between different clusters.
2. Burst‐detection analysis: This analysis aimed to identify citation bursts for specific references. If a citation burst was detected, the corresponding tree ring was colored in red, enabling an examination of the hotspots and research trends during different periods.
3. Dual‐map overlay of citing and cited journals: This method involved overlaying maps of journals that cited the references and journals that were cited. The links between journals represented the pathways of citations, with the thickness of the links indicating the strength of the citations.

### 2.5 Bibliographic analysis using “bibliometrix” R‐package

Bibliometric analysis was conducted using the “bibliometrix” R‐package, which facilitated the counting of national publications in the Web of Science database and the analysis of research collaboration data. Visualization of the results was performed using Scimago Graphica. Additionally, Figure 1 provides an overview of the flowchart of the bibliometric analysis process and presents some of the key findings from this study.

## 3 RESULTS

### 3.1 Section 1. snapshots of PND scientific research

#### 3.1.1 Annual publication number and trend

**FIGURE 2:**
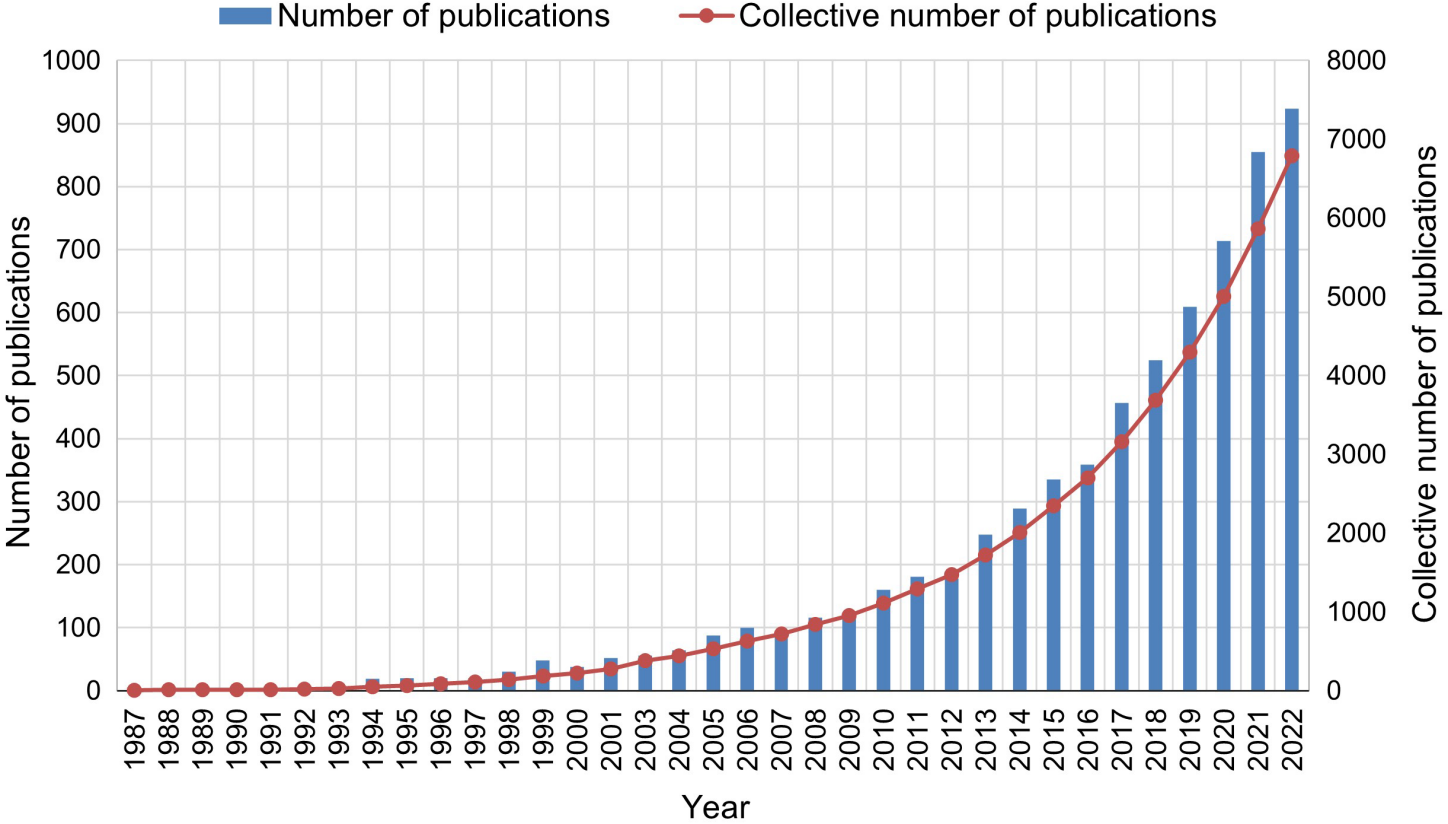
Annual publications.

As previously mentioned, a total of 6787 records related to PND were retrieved from the Web of Science database. Examining the annual trend of research outputs provides valuable insights into the global activity and scientific attention dedicated to POCD. Figure 2 illustrates the time series of published papers on PND. Notably, in the 20th century, the international PND research community did not produce a large number of publications each year, likely reflecting the relatively small number of research groups focused on this topic. However, between 2004 and 2021, there is a clear upward trend in the number of articles published in this research domain, with only 8 publications in 2013. Although the data for 2022 represents only the first half of the year, the count of 50 publications is already remarkable, and it is expected that this year's total number of publications will surpass that of the previous year. Despite only having data for the first half of 2022, the overall publication count continues to grow exponentially.

The first article introducing POD was authored by Morse, RM, and Litin, EM in 1969. From 1969 to 1997, 13 articles, including reports and reviews, focused on POD, highlighting its significance during the early stages of research on POCD. The concept of POCD was introduced by Rasmussen, LS in 1998, in the article “Defining postoperative cognitive dysfunction.” Subsequently, articles primarily addressed POCD and POD. In 2018, Evered, LEvered, L, and Silbert, BSilbert, B et al. published an article titled “Recommendations for the Nomenclature of Cognitive Change Associated with Anesthesia and Surgery‐2018”.^2^ following this publication, the number of annual publications on PND significantly increased.

#### 3.1.2 Contribution by country

**FIGURE 3:**
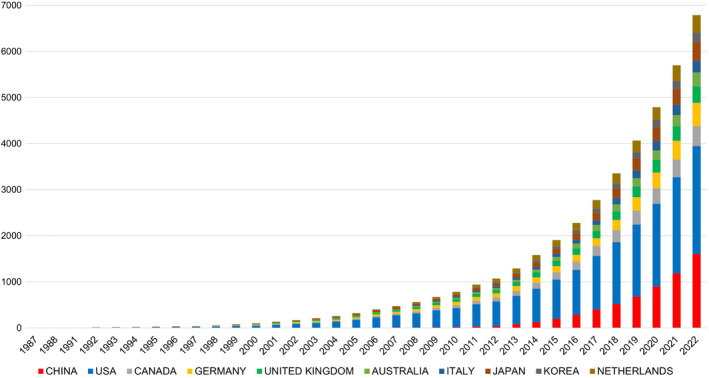
Publications and growth trends.

Regarding the chart depicting the production of publications by countries over time, we identified a total of 6787 articles and reviews on PND published between 1969 and 2022. To ensure clarity and visibility in the chart, the data distribution from 1994 to the first half of 2022 is presented in Figure 3, as the number of published articles during the initial period was relatively small. During this period, the number of original manuscripts addressing PND demonstrated a steady increase, particularly after 2010.

Notably, starting from 2010, China also exhibited a significant interest in PND research and has actively contributed to this area, occupying a prominent position in the research landscape. The overall number of PND publications worldwide has experienced rapid growth. The United States has the highest volume of publications, followed by China, which has shown an increasing proportion in recent years.

#### 3.1.3 Collaboration by country/region

**FIGURE 4:**
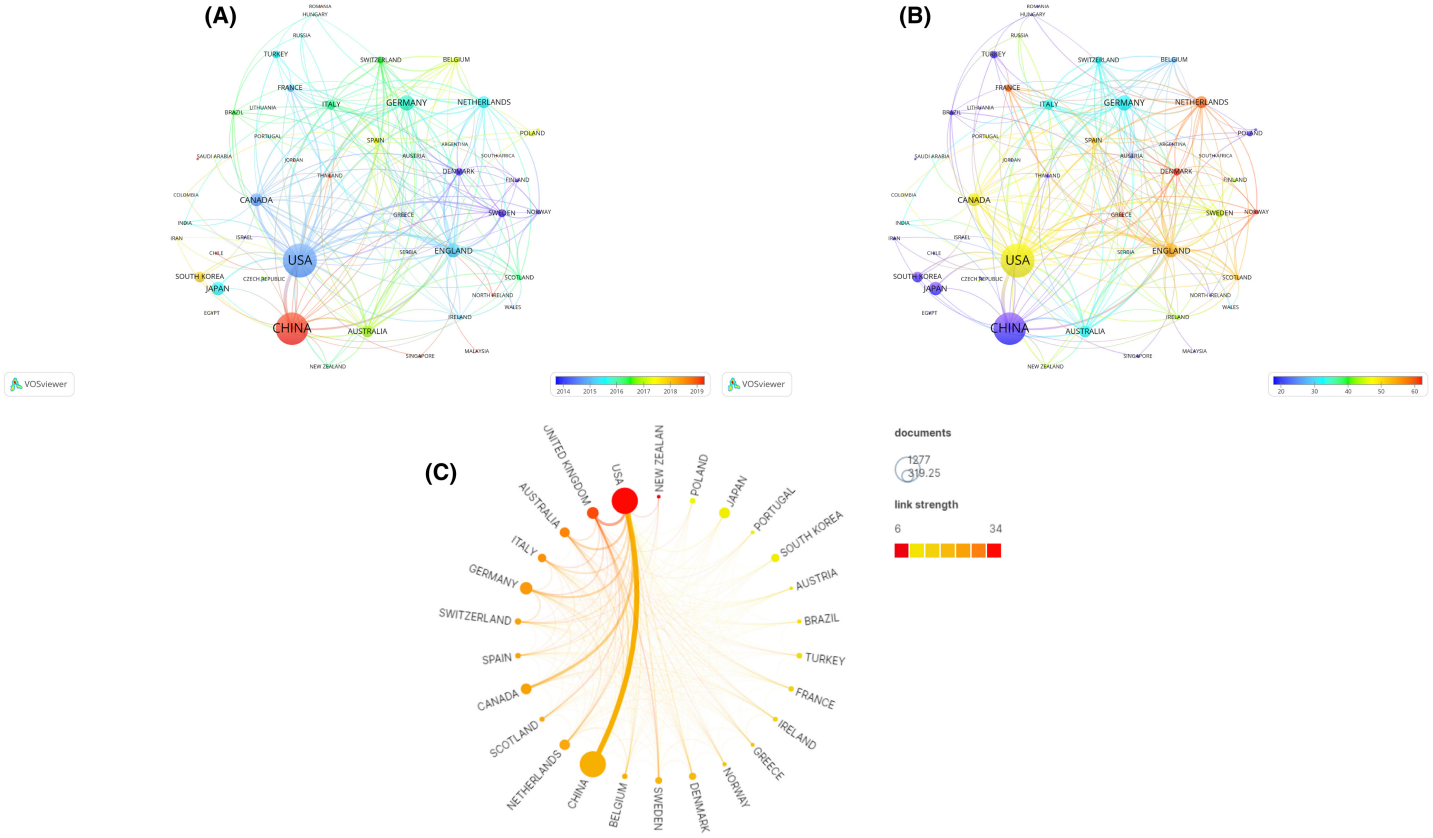
Countries or regions collaboration and network. The size of a node indicates total link strength between countries or regions. The larger the node size, the higher the link strength, that is, the closer collaboration with other countries and regions. The thickness of the links demonstrates the link strength between the two countries or regions. (A) The color of a node indicates average publish year, when the color is closer to purple, the average age of the collaboration with other countries or regions is earlier, conversely, the closer to red, the later the average year of cooperation. (B) The color of a node indicates average citations, when the color is closer to red, the number of average citations with other countries or regions is bigger, conversely, the closer to purple, the fewer the average citations of collaboration. (C) The node represents the number of documents issued by the country, the larger the node, the more the amount of text issued by the country, the closer the node color is to red, and the more the country cooperates with other countries to study the total number of documents. The link indicates the cooperative relationship between countries, and the thicker the link between the two countries, the more times there is cooperation.

The analysis of the country collaboration network indicates that North America and East Asia have emerged as the most vibrant regions in terms of collaborative efforts in recent years. The United States of America and China stand out as the nations fostering the most international partnerships. Notably, the trans‐Pacific affiliation between the USA and China holds substantial prominence. The USA exhibits sturdy academic ties with China, Canada, Australia, Sweden, the Netherlands, and Germany, while the United Kingdom showcases stronger affiliations with fellow European nations. Regarding scholarly output and collaboration intensity, China and the United States forged the most significant alliance. However, it is worth noting that China's collaboration beyond the United States remains relatively limited, which consequently results in a lower overall level of collaborative intensity. In recent years, China has had a large number of publications and has begun to cooperate with European and American countries in PND research, in contrast, the cooperation between the United States and other countries has been carried out earlier and more (Figure 4A).. The collaborative citation endeavors among nations in North America and Europe, wherein the United States exhibit noteworthy publication outputs, are intricately interwoven. However, the collaborative citations between nations in Asia and other countries are relatively low, exhibiting a more nuanced growing trend that warrants further exploration (Figure 4B).

#### 3.1.4 Journals publishing research on the PND

The majority of research articles on PND are published in journals based in both the United States and the United Kingdom. This is likely due to the high prestige and influence of American medical journals as well as UK journals. They are highly regarded platforms for sharing scientific discoveries and innovations, which leads authors from various countries to choose these journals as their publication outlets (Table 1).

**TABLE 1:** Top 20 most published journals in descending order.

Sources/Journal title	Articles	Country
*Anesthesia and Analgesia*	166	The USA
*British Journal of Anaesthesia*	130	The UK
*Anesthesiology*	113	The USA
*Plos One*	108	The USA
*Journal of the American Geriatrics Society*	106	The USA
*BMJ Open*	102	The UK
*Journal of Cardiothoracic and Vascular Anesthesia*	99	The USA
*BMC Anesthesiology*	94	The UK
*Medicine*	90	The USA
*Frontiers in Aging Neuroscience*	82	SWITZERLAND
*Annals of Thoracic Surgery*	79	The USA
*Current Opinion in Anesthesiology*	75	The USA
*Journal of Clinical Anesthesia*	75	The USA
*European Journal of Anaesthesiology*	69	The UK
*International Journal of Clinical and Experimental Medicine*	63	The USA
*Journal of Anesthesia*	59	JAPAN
*Minerva Anestesiologica*	57	ITALY
*Pediatric Anesthesia*	57	The UK
*Anaesthesia*	55	The UK
*Acta Anaesthesiologica Scandinavica*	46	DENMARK

### 3.2 Section 2. the history and development of PND

#### 3.2.1 Scientific categories

Each journal in the WOSCC is categorized into specific scientific disciplines, providing valuable insights into the domains and disciplines with which the journals are associated. Analyzing the distribution of these categories across the entire dataset offers a glimpse into the positioning of the PND domain within the broader scientific knowledge landscape.

**FIGURE 5:**
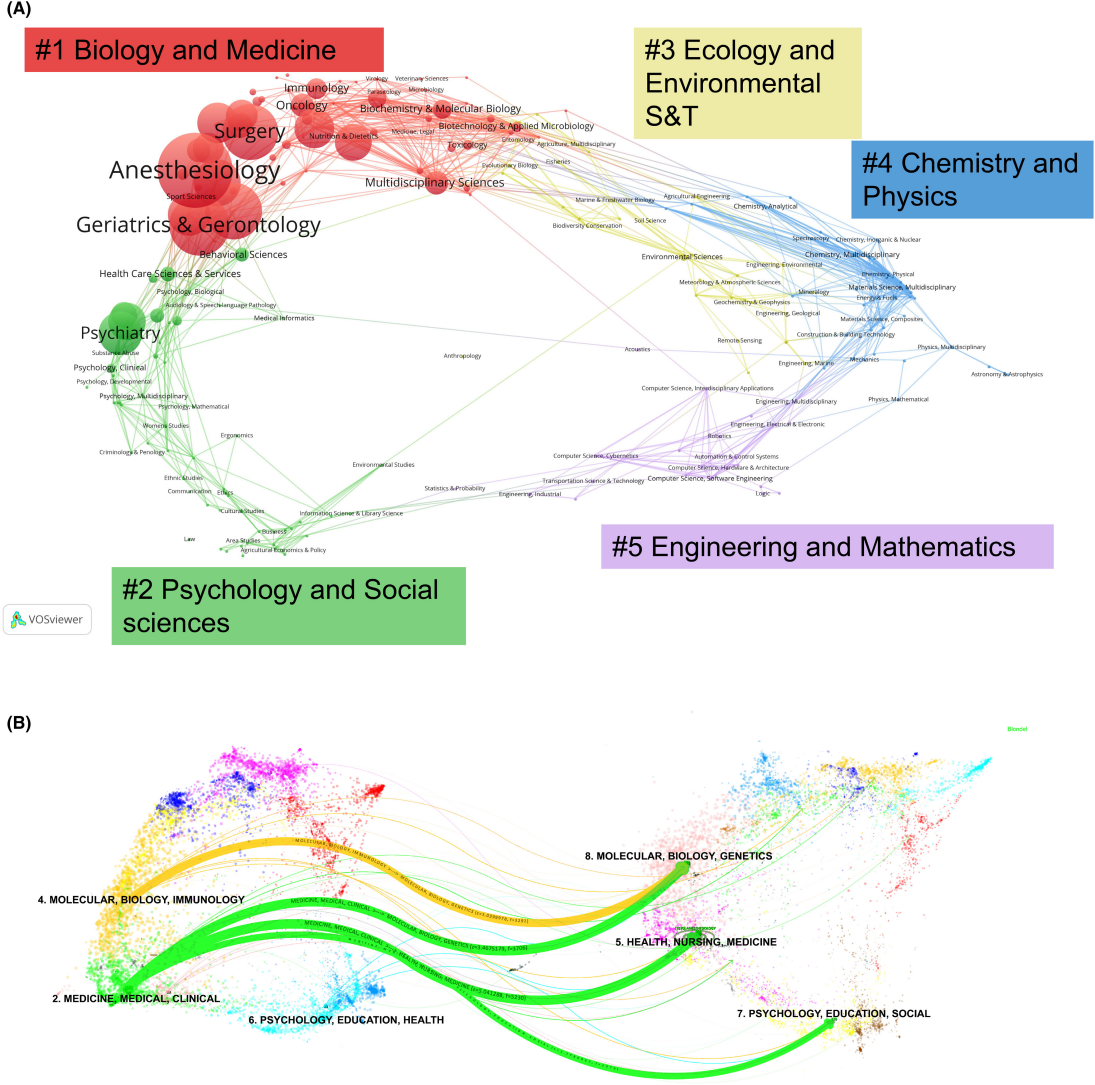
Scientific categories and dual‐map overlay. (A) Scientific categories. (B) Labeled ovals are used to indicate clusters of highly active citing and cited journals. The size of the ovals is proportionate to the number of publications for the citing journals on the left and to the number of citations received from PND articles by a journal on the right. On the left‐hand side of the Figure 5B, the distribution of PND journals on the global science map is shown, whereas the right‐hand side shows the distribution of cited journals, and the curve is the quotation association line from the outside to the relevant side.

The distribution of scientific categories associated with PND is illustrated in the form of a global science map,^5^ drawn using the VOSviewer software.^6^ The global scientific categories are grouped in five clusters (Figure 5A), such as #1 “Biology and Medicine”, #2 “Psychology and Social Sciences”, #3 “Ecology and Environmental Science and Technology”, #4 “Chemistry and Physics”, and #5 “Engineering and Mathematics”. The results reveal that PND research primarily falls within the “Biology and Medicine” domain (cluster #1). Additionally, the “Psychology and Social Sciences” domain (cluster #2) emerges as the second most prominent area. This visualization not only highlights the key contributing scientific categories but also emphasizes the interdisciplinary nature of the PND research domain.

#### 3.2.2 Journals’ distribution and intellectual base

The dual‐map overlay analysis was conducted using CiteSpace,^7^ and the journal‐based dual‐map overlay was created following the approach described by Carley and colleagues.^8^ This analysis enables the visualization of journals from a specific dataset on the global science map of journals. The analysis involves tracing the cited journals in the reference lists of these journals, placing them on a separate journal overlay map, and connecting both maps. This connection illustrates the flow of knowledge and the relationships between different research areas. In the context of PND, the analysis reveals that articles are predominantly published in journals categorized under “Medicine, Medical, Clinical” (Figure 5B). The cited journals, which can be considered the intellectual foundation of the research domain, are primarily clustered within the “Health, Nursing, Medicine,” “Psychology, Education, Social,” and “Molecular, Biology, Genetics” journal groups. It is noteworthy that the majority of citing journal groups reference journals from the “Medicine, Medical, Clinical” group, a significant portion of citing journal groups reference journals from the “Molecular, Biology, Immunology”, while a smaller set of journal domains, predominantly related to “Psychology, Education, Social.” This observation suggests that despite some degree of interdisciplinarity, the intellectual basis of PND research remains relatively concentrated within specific scientific subdomains.

### 3.3 Section 3. current and future research themes

#### 3.3.1 Co‐citation analysis

**FIGURE 6:**
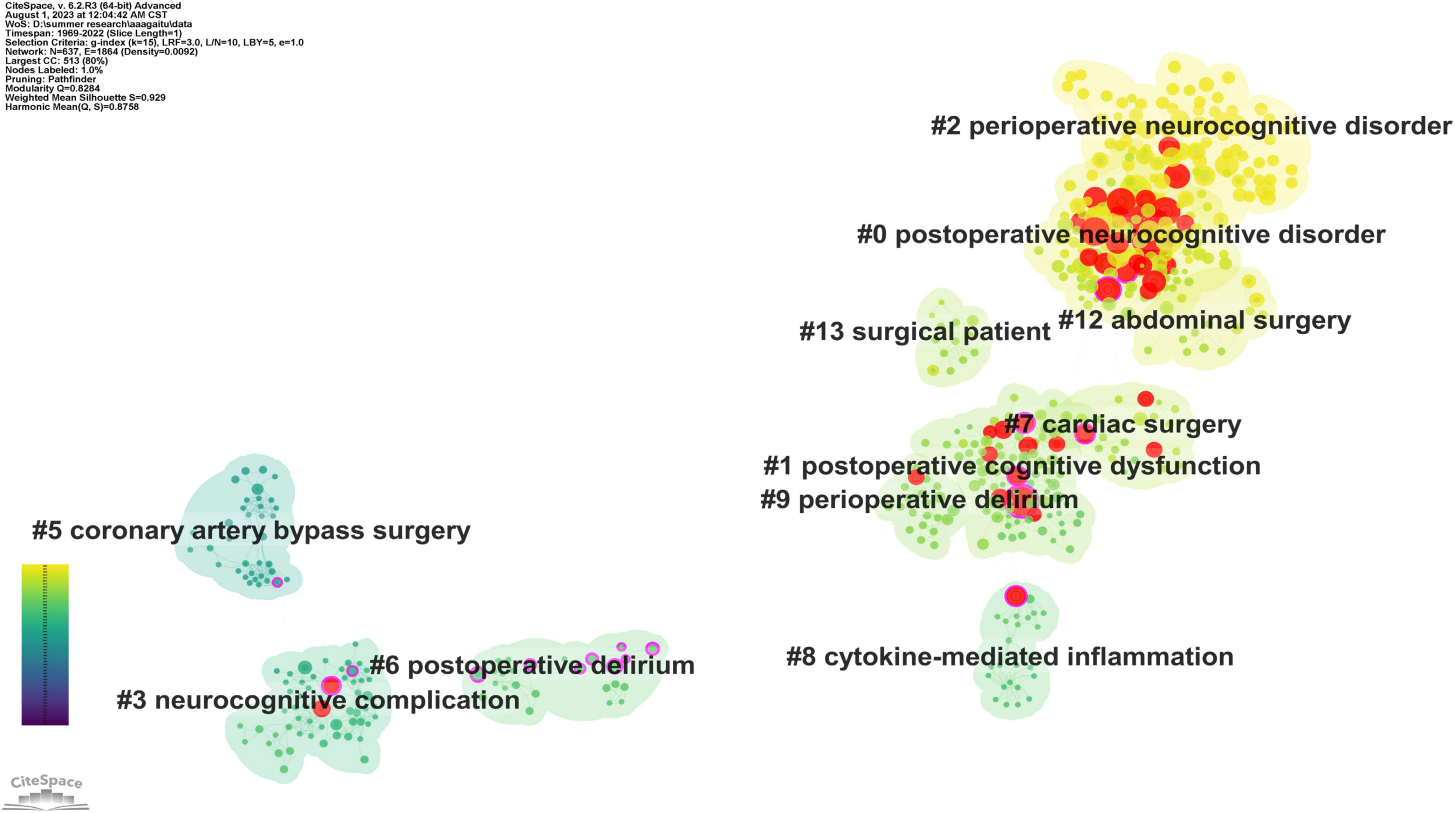
Cited Reference cluster. In the figure, the node sizes are proportional to the number of citations of a publication, while the colors of the links between articles indicates the year when two documents were first cited together. The color shade of the clusters indicates the average publication year of the references. The size of a node indicates how many citations the associated reference received. Each node is depicted with a series of citation tree‐rings across the series of time slices. The structural properties of a node are displayed in terms of a purple ring. The thickness of the purple ring indicates the degree of its betweenness centrality, which is a measure associated with the transformative potential of a scientific contribution. Such nodes tend to bridge different stages of the development of a scientific field. Citation rings in red indicate the time slices in which citation bursts, or abrupt increases of citations, are detected.

In this section, we employed CiteSpace^7^, ^9^, ^10^ to establish a coherent structure of the obtained results. Co‐citation analysis was conducted on the complete dataset timespan (1969–2022), employing a time slice length of 1 year, a five‐year look‐back period for considering cited references, and a minimum requirement of two citations per period. The resulting co‐citation network consists of 637 nodes and 1864 co‐citation links. The largest connected component of this network is illustrated in Figure 6.

In our study, the clustering analysis revealed a high level of clarity and reliability in the structure, as indicated by Modularity *Q* = 0.828 (>0.3) and Mean Silhouette = 0.929 (>0.7). The primary findings from the co‐citation analysis of the largest connected cluster network are presented in Table 2. Notably, the clusters of “postoperative neurocognitive disorder”(0^#^ cluster), “postoperative cognitive dysfunction” (1^#^, 2^#^, and 12^#^ cluster), and “post‐or perioperative delirium” (6^#^, 7^#^, 9^#^, and 17^#^ cluster) emerged as the most prominent.

**TABLE 2:** Top 10 largest co‐citation clusters.

Cluster ID	Size	Silhouette	Label (LSI)	Label (LLR)	Label (MI)	Avg (YR)	Research Front (DOI)
0	125	0.917	Postoperative neurocognitive disorder	Intraoperative hypotension (63.84, 1.0E‐4)	Surgery‐hyperthermic intraperitoneal chemotherapy (4.57)	2016	10.1111/cns.13873
1	90	0.829	POCD	Old mice (162.6, 1.0E‐4)	Surgery‐hyperthermic intraperitoneal chemotherapy (0.48)	2010	10.1111/anae.12493
2	77	0.913	POCD	PND (92.98, 1.0E‐4)	Anesthetic management (1.77)	2018	10.3389/fncel.2022.843069
3	55	0.98	Neurocognitive complication	Neurocognitive complication (58.57, 1.0E‐4)	POCD (0.07)	2001	10.1002/ana.20481
5	35	0.99	Coronary artery bypass surgery	Neurocognitive dysfunction (37.83, 1.0E‐4)	POCD (0.08)	1996	10.1067/mtc.2000.108901
6	25	0.992	POD	Intensive care unit (40.62, 1.0E‐4)	POCD (0.09)	2005	10.1016/j.ccc.2007.10.002
7	24	0.984	POD	Cardiac surgery patient (75.15, 1.0E‐4)	Hippocampal autophagy (0.11)	2012	10.1053/j.jvca.2014.08.021
8	24	0.996	Cytokine‐mediated inflammation	Cytokine‐mediated inflammation (56.3, 1.0E‐4)	POCD (0.08)	2005	10.1097/00000542‐200,703,000‐00007
9	21	0.939	Perioperative delirium	Perioperative delirium (61.95, 1.0E‐4)	POCD (0.07)	2009	10.1016/j.pnpbp.2012.11.005
12	13	0.976	POCD	Abdominal surgery (51.2, 1.0E‐4)	POCD (0.07)	2014	10.1016/j.bbi.2016.02.003

Abbreviations: Avg (YR), the average publication year of the references in the cluster; Label (LLR), log‐likelihood ratio; Label (LSI), Latent Semantic Indexingn; Label (MI), mutual information MI; Research Front, article which cited most papers from the cluster; Size, number of publications in the cluster.

The examination of cluster landscapes and their temporal evolution revealed that earlier research frontiers in PND investigation revolved around “cognitive decline” and “coronary artery bypass surgery.”^11^ This observation suggests that the exploration of PND originated from the recognition of cognitive decline among individuals following specific surgeries and anesthesia. Subsequently, multiple research clusters have endeavored to elucidate the elevated mortality and morbidity associated with undergoing surgery under anesthesia.

One possible mechanism, centered on long‐term alterations of neurotransmitters following general anesthesia but with less impact after regional anesthesia, has been proposed. Subsequently, numerous research clusters have emerged, aiming to enhance our understanding of the mechanisms underlying PND and identify independent predictors of delirium. Given that POD and POCD are the primary cognitive disorders observed after surgery, the majority of PND research clusters have concentrated on these conditions. These clusters have specifically focused on clinical risk management and cognitive protection throughout the evolution of the research domain, including perioperative risk management, perioperative psycho‐education intervention, and perioperative cognitive protection.

By analyzing the most relevant sources cited within each cluster, we can gain valuable insights into their respective focal points. For instance, the largest cluster comprises 125 members, and the most significant source cited within this cluster is a narrative review authored by Hao Kong (2022). This comprehensive narrative review provides insights into the diagnosis, prevention, and treatment of PND, rendering it the primary source cited within cluster #0.

#### 3.3.2 Highly cited papers’ analysis

To enrich our understanding of highly cited papers, we have dissected those articles that have greatly contributed to the advancement of this field (Table 3., Figure 8). A significant part of clinical work has pivoted around identifying perioperative interventions for PND, which could potentially mitigate postoperative cognitive impairment. In 2008, Monk et al. determined that factors such as increasing age, lower educational attainment, a prior occurrence of cerebral vascular accident devoid of lasting impairment, and POCD evident upon hospital discharge, were all autonomous risk factors for cognitive decline observed 3 months after major noncardiac surgery,^12^ and results from a large cohort study conducted by Daiello et al., which followed up for a median of 8.50 years, showed that cognitive dysfunction after noncardiac surgery was associated with increased mortality, risk of leaving the labor market prematurely, and dependency on social transfer payment.^13^

**TABLE 3:** The top 10 ranked item by citation counts.

Citation counts	Reference	DOI	Cluster ID
58	Evered L, 2018, BRIT J ANAESTH, V121, P1005	10.1016/j.bja.2017.11.087	0
34	Evered LA, 2018, ANESTH ANALG, V127, P496	10.1213/ANE.0000000000003514	0
34	Aldecoa C, 2017, EUR J ANAESTH, V34, P192	10.1097/EJA.0000000000000594	0
30	Skvarc DR, 2018, NEUROSCI BIOBEHAV R, V84, P116	10.1016/j.neubiorev.2017.11.011	0
29	Needham MJ, 2017, BRIT J ANAESTH, V119, PI115	10.1093/bja/aex354	0
27	Daiello LA, 2019, ANESTHESIOLOGY, V131, P477	10.1097/ALN.0000000000002729	0
25	Steinmetz J, 2009, ANESTHESIOLOGY, V110, P548	10.1097/ALN.0b013e318195b569	1
25	Inouye SK, 2016, ALZHEIMERS DEMENT, V12, P766	10.1016/j.jalz.2016.03.005	0
24	Feng XM, 2017, JCI INSIGHT, V2, P0	10.1172/jci.insight.91229	0
21	Subramaniyan S, 2019, ANESTH ANALG, V128, P781	10.1213/ANE.0000000000004053	2

Later in 2012, a prospective study by Saczynski et al.^14^ examining patients undergoing cardiac surgery revealed that delirium was correlated with a considerable decrement in cognitive ability within the first year post‐cardiac surgery. This pattern was defined by an immediate drop and a sustained impairment, signifying that the onset of delirium post‐surgery posed a risk for cognitive function decline and extended periods of impairment after surgery. Consequently, delirium prevention should be a matter of priority.

In 2014 and 2016, Inouye et al.^15^, ^16^ published a systematic review entitled “Delirium in Elderly People” published in the Lancet. The review not only elucidated that delirium is prevalent, severe, costly, and often undetected in elderly individuals and that there is no compelling evidence of the efficacy of pharmacological prevention or treatment methods. Rather, reducing sedative and analgesic medications and utilizing nonpharmacological approaches were suggested. The review underscored that multicomponent nonpharmacological risk factor interventions were the most effective preventive strategy. It further emphasized that delirium presents an opportunity to shed light on brain pathophysiology‐acting as an indicator of a vulnerable brain with a decreased reserve and as a possible mechanism for enduring cognitive damage. While several biomarkers related to delirium have been pinpointed, the underlying pathophysiological basis remains unclear, leaving crucial knowledge gaps to be filled. Inouye et al. also probed the association between delirium and long‐term cognitive decline, establishing that individuals with delirium exhibited a significant decrease in cognition below baseline levels beyond 36 months at a rate akin to that of mild cognitive impairment. Subsequently, in 2017, Aldecoa et al.^17^ proposed guidelines outlining evidence‐based and consensus‐based recommendations for the prevention and management of POD. These guidelines stressed the importance of preoperative identification and management of patients at risk, proper intraoperative care, POD detection, and the handling of patients with delirium.

Given the multitude of medical specialties involved in the care of surgical patients, it is crucial to adopt a team‐based approach in daily clinical practice. Consequently, this guideline has gained substantial recognition across various disciplines, effectively enhancing knowledge and education in the preoperative, intraoperative, and postoperative settings. Its impact extends beyond anesthesiologists to encompass all healthcare professionals responsible for the well‐being of surgical patients.

**FIGURE 7:**
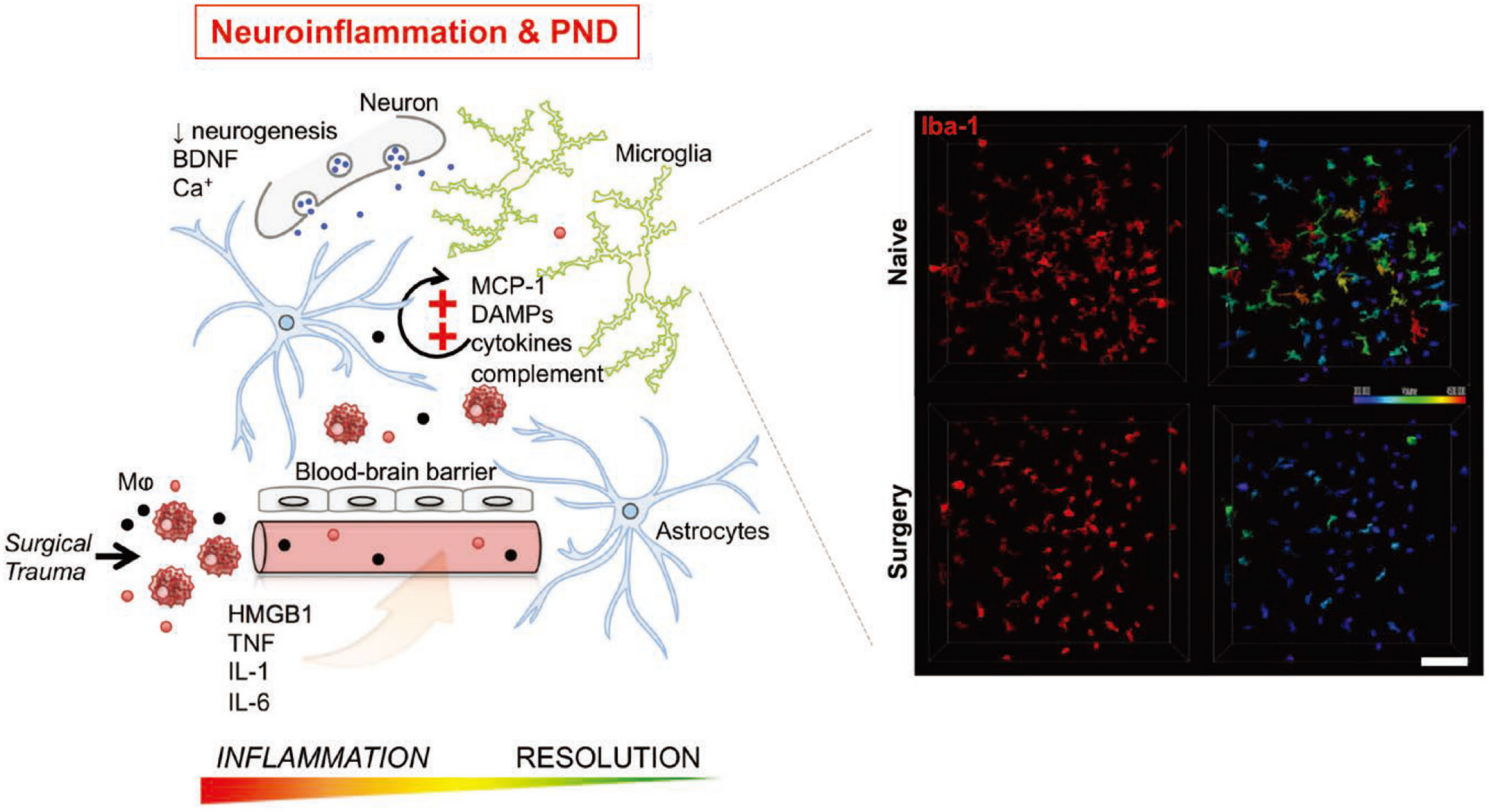
Schematic overview mechanisms involved in postoperative neuroinflammation work. *Quoted from Subramaniyan et al.'s “Schematic overview of mechanisms involved in postoperative neuroinflammation work”. Published in Anesthesia & Analgesia. 2019 Apr;128(4):781–8.

Researchers have dedicated significant efforts to studying the pathophysiological mechanisms underlying POCD. Accumulating evidence suggests that neuroinflammation may play a pivotal role in this process, as evidenced by the presence of proinflammatory signaling molecules in both patients and animal models of PND. Dysregulated inflammation and neuronal injury have emerged as crucial factors in PND based on clinical studies. Presently, the mechanism of neuroinflammation has become a focal point of POCD research, with particular emphasis on the innate immune response triggered by aseptic surgical trauma and subsequent neuroinflammation.^18^, ^19^ Limiting the acute neuroinflammatory impact of surgery could potentially preserve cognitive function and significantly improve the risk–benefit profile of surgical interventions. In 2017, Feng et al.^20^ demonstrated that depleting microglia during the perioperative period provided remarkable protection against POCD in mice. This protection was associated with reduced levels of inflammatory mediators in the hippocampus, suppression of CCR2^+^ leukocyte recruitment in the hippocampus, and increased levels of circulating inflammation‐resolving factors. Thus, targeting microglia could be a viable strategy to mitigate the development of POCD, particularly in individuals with heightened vulnerability. In 2019, Subramaniyan et al.^21^ conducted a comprehensive review focusing on the role of postoperative neuroinflammation and the underlying mechanisms involved in immune‐to‐brain signaling following peripheral surgery (Figure 7). Surgical procedures activate the innate immune system, leading to the release of proinflammatory mediators, such as cytokines, chemokines, alarmins, and eicosanoids, as well as the activation of systemic immunocompetent cells. These processes adversely affect the integrity of the blood–brain barrier, resulting in endothelial dysfunction and the infiltration of peripheral cells and factors into the brain parenchyma. Within the central nervous system, astrocytes and microglia transition from their resting state and contribute to overall neuronal dysfunction and impaired memory function. Initiating resolution programs early during the inflammatory response may offer neuroprotective strategies against POCD. The role of inflammation in perioperative brain function is increasingly apparent, prompting a growing body of preclinical investigations aimed at better comprehending the impact of surgery and anesthesia on the central nervous system and their potential contributions to cognitive decline.

In 2016, Su et al.^22^ were the first to propose that the preventive administration of a low‐dose of dexmedetomidine, a sedative that acts as an α_2_ adrenoceptor agonist, significantly reduces the incidence of POD without an associated increase in adverse events. Nevertheless, it remains unclear whether the beneficial effects observed with this innovative utilization of dexmedetomidine translate into improved long‐term outcomes.

In 2017, Needham et al. pointed that recent articles have highlighted the difficulties of confirming any clear links between anesthesia and cognitive dysfunction, in part because of the lack of consistency regarding definition and diagnosis. So clinical research studies are frequently confounded by a lack of agreed definitions and consistency of testing.^23^ With the ongoing convergence of the multidisciplinary approach to PND and the continuous advancements in brain science, there is a growing trend toward harmonizing the assessment methods and processes used by various disciplines, such as geriatrics, neurology, surgery, and anesthesia. These disciplines have adopted similar terminology to the cognitive classifications utilized in general population studies when investigating cognitive changes following anesthesia and surgery. In 2018, Evered et al.^24^, ^25^ a working group comprising experts from multiple specialties, recommended the use of “perioperative neurocognitive disorders” as a comprehensive term encompassing cognitive impairment or changes identified in the preoperative or postoperative period. This terminology encompasses cognitive impairment diagnosed prior to the operation, any form of acute cognitive event (e.g., delirium), and cognitive decline diagnosed within 30 days after the procedure (referred to as delayed neurocognitive recovery) and up to 12 months.^26^ The efforts of Evered et al. significantly contributed to our understanding of cognitive decline following anesthesia and surgery by establishing consistent terminology that extends beyond the fields of anesthesia and surgery.^24^ This unification enables research conducted in geriatrics, geriatric psychiatry, and neurology to directly apply to the context of anesthesia and surgery. Consequently, the concept of POCD is being phased out from the lexicon, making way for terms that hold direct clinical significance.

#### 3.3.3 Citation burst analysis

**FIGURE 8:**
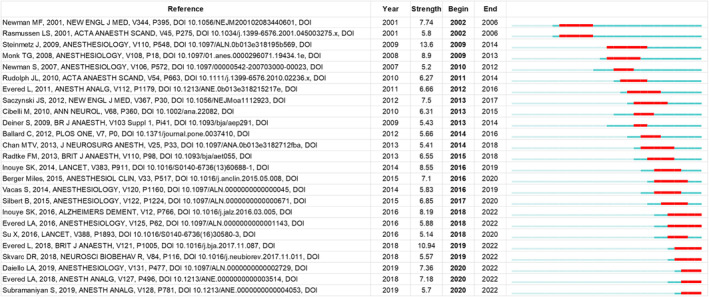
Top 25 Reference.

The concept of citation burst is a powerful tool for identifying rapidly evolving ideas and prospective research issues within a given field. This method proves effective for assessing emergent trends and unexpected shifts in the advancement of a discipline, thereby highlighting areas of active research or those on the cutting edge. Therefore, it is a valuable approach for tracking shifts in research emphasis. An examination of the top 25 references reveals that over the past two decades, scholars have given considerable attention to research on PND (Figure 8). Concurrently, it has been observed that most of the articles cited in Table 3 garnered the majority of their citations within 3–5 years following publication. This observation indicates that PND research undergoes frequent updates and iterations. A study of citation burst analysis revealed that current investigations primarily focus on the development of improved clinical management practices aimed at preventing POD and long‐term cognitive decline. Many studies were focused on POD because POD significantly increased the risk of POCD in the first postoperative month. Delirium and POCD shared risk factors and may co‐occur, although their relationship is unclear.^13^ Persons with delirium had significantly greater long‐term cognitive decline relative to the nondelirium group.^16^ Considering that delirium is preventable, it is essential to emphasize the significance of preventive measures in reducing its immediate and potential long‐term negative consequences. Moreover, previous results hold implications for clinical trials, as treatment during the acute phase where most patients will recover, may not necessarily lead to long‐term benefits; thus, new interventions may be required to forestall long‐term cognitive decline after delirium. Many researchers were constantly exploring and confirming the inflammatory response and oxidative stress following surgery contributed to the pathophysiology of POCD by impairing neuronal function and promoting neuroinflammation in the CNS, discussing the potential use of various drugs, such as statins, pregabalin, dexmedetomidine, and minocycline, in preventing or treating cognitive impairment. However, further research is needed to fully understand the effectiveness of these interventions and their impact on cognitive function.^27^ Additionally, there is a significant emphasis on the standardized implementation of research pertaining to cognitive function and the formalized terminology associated with perioperative cognitive decline.

#### 3.3.4 Keyword or term analysis

**FIGURE 9:**
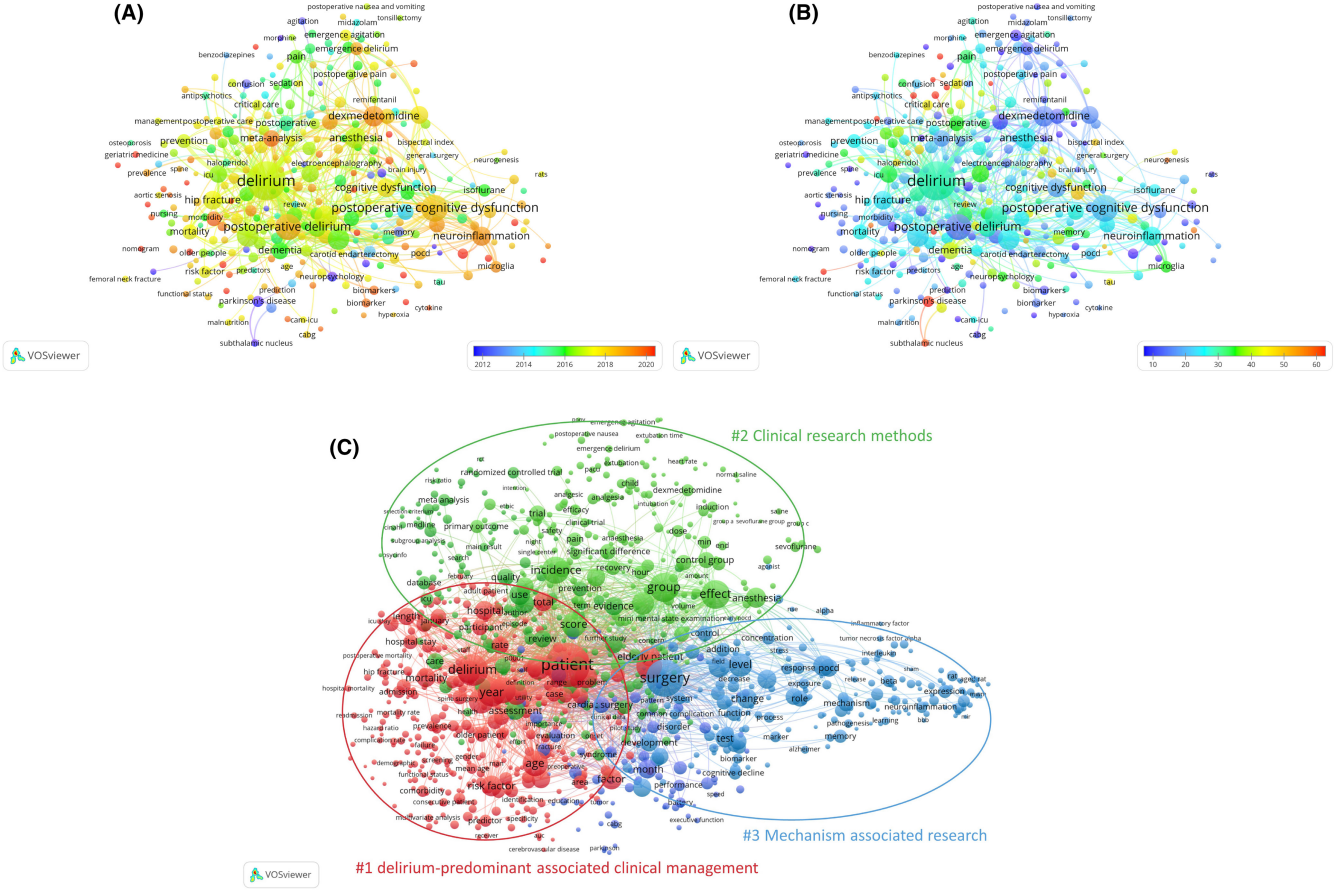
Keywords and terms on PND. The node size of the keywords is associated with the keyword occurrences. The larger the node size, the higher the occurrence frequency. (A) If the color of the node is closer to purple, the average published year is earlier, and the closer to red, the later. (B) If the color of the node is closer to purple, the number of average citations is fewer, and the closer to red, the higher citations. (C) The node size of the keywords is associated with the keyword occurrences. The larger the node size, the higher the occurrence frequency.

The keyword and term analysis revealed that PND research encompasses a broad range of fields beyond anesthesiology, including geriatrics, neuroscience, psychiatry, pharmacology, and more (Table 4). Examination of highly occurring keywords identified “delirium” and “aged” as prominent topics within PND research. Based on word frequency, three main term clusters were identified: the first term cluster focuses on the clinical management of delirium and its associated factors, which encompasses various aspects such as prevention, assessment, and treatment strategies for delirium in different patient populations; the second term cluster highlights research related to POCD, which includes studies investigating risk factors, underlying mechanisms, diagnostic criteria, and interventions aimed at mitigating cognitive decline following surgery; the third term cluster centers around clinical research methods and methodologies used in studies related to delirium and POCD, which may involve discussions on study design, statistical analyses, outcome measures, and data collection techniques.(Figure 9).

**TABLE 4:** Main categories chart from Web of Science.

Categories	Records	Percentage
Anesthesiology	1302	19.184
Surgery	1149	16.929
Clinical Neurology	763	11.242
Geriatrics Gerontology	763	11.242
Neurosciences	713	10.505
Medicine General Internal	615	9.061
Cardiac Cardiovascular Systems	552	8.133
Psychiatry	412	6.070
Medicine Research Experimental	405	5.967
Pharmacology Pharmacy	349	5.142
Respiratory System	342	5.039

## 4 DISCUSSION

In this article, we conducted a scientific econometric analysis of PND. Utilizing the WOS database as our data source, we applied various scientific and quantitative methods, along with visualization tools, to explore key aspects such as publication trends over time, national publication volume, national and regional collaborations, and terminology analysis. Examining the timeline of publications, we observed that research in the field of PND originated in 1969 and has since experienced exponential growth. Particularly, after 2000, there has been a noticeable surge in attention toward PND. In terms of national publication volume, the United States has been a prominent contributor to PND literature over the past two decades, while China has witnessed a significant increase in the number of PND articles in recent years. Analyzing collaboration networks among countries and regions, we observed a close and frequent partnership between China and the United States. Through the analysis of terms, we identified three major research clusters in the field of PND: delirium‐predominant associated clinical management, POCD research, and clinical research methods. Key keywords in PND research include POD and POCD. Based on scientific categorization, PND research is primarily concentrated within the domains of “Biology and Medicine” and “Psychology and Social Sciences.” As anesthesia research progresses, we anticipate a growing number of PND articles highlighting the significance of PND in the field. Additionally, with the international definition of PND, future research will likely expand into diverse areas. PND research will find wider application in exploring cognitive changes in surgical and non‐surgical populations, facilitating investigations into the mechanisms underlying PND and brain function decline. Ultimately, this research will assist healthcare professionals in developing effective strategies for the prevention, identification, and management of PND.

### 4.1 Strengths and limitations

Despite employing a rigorous approach, it is crucial to acknowledge certain limitations of this study. Firstly, we focused exclusively on research articles within the WOSCC and excluded other types of publications, such as letters and conference articles. As a result, the comprehensiveness of the analysis may have been constrained. Secondly, the presence of identical author name abbreviations in articles posed a challenge for bibliometric software, which could not accurately distinguish between contributions from authors with the same name. Although efforts were made to address this issue, some degree of accuracy loss may still be unavoidable. Thirdly, while we identified the main research contributions from the literature, the analysis may still be incomplete, and further examination of the literature is necessary to explore additional meaningful research directions. Fourthly, the review specifically focused on articles with an available English abstract obtained from online databases. While English serves as the predominant language in most journals and abstracts are commonly accessible, this selection criterion may introduce a language and publication bias.

## 5 CONCLUSION

This study highlights the rapid growth of research on PND in recent years. This research provided an overview of previous studies in the field of PND, thereby establishing the overall landscape of PND research and identifying potential avenues for future investigations. Moreover, with the aging population undergoing surgery in larger numbers, there is a significant presence of individuals experiencing cognitive impairment either before or after undergoing surgical anesthesia. Perioperative, medical practitioners must be equipped to understand and manage these patients. It is crucial to promote multidisciplinary collaboration throughout the perioperative period for future clinical studies.

### AUTHOR CONTRIBUTIONS

Duan Gao: data collection, data analysis, and writing – original draft preparation. Ning Yang: conceptualization, writing – reviewing and editing, and supervision. Yu Qiao: methodology, software, and Validation. Ruoxuan Liu: data collection, data analysis, and writing – original draft preparation. Zihang Zhang: data collection and visualization. Mingzhang Zuo: co‐corresponding author. Who response for the manuscript's submission, revisions, and communication with the publication venue.

## CONFLICT OF INTEREST STATEMENT

All authors have approved the manuscript for submission and without any potential competing interests.

## ETHICS STATEMENT

This study did not involve human participants, human data, or human tissue, nor did it involve the use of animals. Therefore, it did not require ethical approval in accordance with local guidelines and regulations. The research was conducted adhering to the highest standards of integrity and academic honesty. Throughout the study, all applicable institutional and national guidelines for the care and use of materials were followed.
